# Determinants of infant growth in Eastern Uganda: a community-based cross-sectional study

**DOI:** 10.1186/1471-2458-8-418

**Published:** 2008-12-22

**Authors:** Ingunn Marie Stadskleiv Engebretsen, Thorkild Tylleskär, Henry Wamani, Charles Karamagi, James K Tumwine

**Affiliations:** 1 Centre for International Health, University of Bergen, Bergen, Norway; 2 Makerere University School of Public Health, Kampala, Uganda; 3 Makerere University Clinical Epidemiology Unit, Kampala, Uganda; 4 Department of Paediatrics and Child Health, Makerere University, Kampala, Uganda

## Abstract

**Background:**

Child under-nutrition is a leading factor underlying child mortality and morbidity in Sub-Saharan Africa. Several studies from Uganda have reported impaired growth, but there have been few if any community-based infant anthropometric studies from Eastern Uganda. The aim of this study was to describe current infant growth patterns using WHO Child Growth Standards and to determine the extent to which these patterns are associated with infant feeding practices, equity dimensions, morbidity and use of primary health care for the infants.

**Methods:**

A cross-sectional survey of infant feeding practices, socio-economic characteristics and anthropometric measurements was conducted in Mbale District, Eastern Uganda in 2003; 723 mother-infant (0–11 months) pairs were analysed. Infant anthropometric status was assessed using z-scores for weight-for-length (WLZ), length-for-age (LAZ) and weight-for-age (WAZ). Dependent dichotomous variables were constructed using WLZ < -2 (wasting) and LAZ < -2 (stunting) as cut-off values. A conceptual hierarchical framework was used as the basis for controlling for the explanatory factors in multivariate analysis. Household wealth was assessed using principal components analysis.

**Results:**

The prevalences of wasting and stunting were 4.2% and 16.7%, respectively. Diarrhoea during the previous 14 days was associated with wasting in the crude analysis, but no factors were significantly associated with wasting in the adjusted analysis. The adjusted analysis for stunting showed associations with age and gender. Stunting was more prevalent among boys than girls, 58.7% versus 41.3%. Having brothers and/or sisters was a protective factor against stunting (OR 0.4, 95% CI 0.2–0.8), but replacement or mixed feeding was not (OR 2.7, 95% CI 1.0–7.1). Lowest household wealth was the most prominent factor associated with stunting with a more than three-fold increase in odds ratio (OR 3.5, 95% CI 1.6–7.8). This pattern was also seen when the mean LAZ was investigated across household wealth categories: the adjusted mean difference between the top and the bottom wealth categories was 0.58 z-scores, p < 0.001. Those who had received pre-lacteal feeds had lower adjusted mean WLZ than those who had not: difference 0.20 z-scores, p = 0.023.

**Conclusion:**

Sub-optimal infant feeding practices after birth, poor household wealth, age, gender and family size were associated with growth among Ugandan infants.

## Background

Child under-nutrition is a leading factor underlying child mortality and morbidity in Sub-Saharan Africa, and contributes to 2.2 million deaths and a fifth of all disability-adjusted-life-years lost worldwide for children under five years old [1]. Early initiation is an important contributor to successful breastfeeding, and breastfeeding is an effective way of reducing neonatal mortality in resource-deprived settings [2]. Breastfeeding interventions have a huge effect on neonatal and infant health, but it is challenging to implement and sustain such programmes successfully [3]. Multi-dimensional interventions are needed to prevent different aspects of nutritional depletion, e.g. severe malnutrition and stunting. Nutritional programmes should focus on sufficient feeding for individuals as well as families, community work, health care delivery systems and other underlying determinants, including poverty [4]. There is a need to focus on the youngest children in nutrition programmes to prevent long-term effects [5].

Several anthropometric studies in Uganda have described impaired linear growth among children up to five years old. Stunting (length/height-for-age less than -2 z-scores) occurs in 25% of children under two years [6,7] and in 50% of children up to five years [8,9]. It has been emphasized that this pattern can already be detected in infancy and that the process starts *in utero *[10]. There have been few community studies on infant growth patterns in Uganda, and to our knowledge, none in Eastern Uganda. Our study was a pilot survey in the ongoing study: 'Promoting infant health and nutrition in Sub-Saharan Africa: Safety and efficacy of exclusive breastfeeding promotion in the era of HIV (PROMISE EBF)' (Id: NCT00397150 at ), which is a cluster-randomized multi-centre trial of the safety and efficacy of exclusive breastfeeding (EBF) promotion by peer-counsellors among both HIV-1 infected and uninfected mothers in Burkina Faso, Zambia, South Africa and Uganda [11]. The aim of our study was to describe infant growth patterns including weight-for-height, height-for-age and weight-for-age in relation to equity dimensions, infant feeding practices, morbidity and primary health care usage in Eastern Uganda, assessed using the WHO Child Growth Standards [12].

## Methods

### Study site, sampling and participants

The study was conducted from September to November 2003 in Mbale District, Eastern Uganda, in one urban area (Mbale municipality) and the surrounding rural county of Bungokho. Administrative information was retrieved from the Uganda Bureau of Statistics in Entebbe, which gave us parish sizes and the number of villages within each parish . The populations were sampled on the basis of probability proportional to size: the appropriate number of villages was randomly selected in each parish according to parish size and seven households were randomly selected in each village. The sample was not stratified on urban/rural status. The study site, design, questionnaire details and definitions are fully described elsewhere [13]. A national programme for the prevention of mother-to-child transmission of HIV-1 (PMTCT) was launched at Mbale Regional Referral Hospital in 2000, but the success rate was described as low in 2003, with less than 10% utilisation of voluntary counselling and testing (VCT) [14]. The population is semi-urban and comprises mainly subsistence farmers. We contacted 793 randomly-selected caretaker-infant (0–11 months) pairs; 30 were non-respondents, and 36 were excluded because the caretaker was not the mother of the infant and the data were incomplete. Four were excluded because of inconsistent anthropometric values. The exclusion criteria we used were weight-for-length z-scores (WLZ) more than +2 and length-for-age z-scores (LAZ) less than -3. This was in line with a conservative interpretation of the criteria used by WHO to avoid "unhealthy weights for length/height, observations falling above +3 SD (standard deviations) and less than -3 SD" [15]. We excluded no infants with WLZ less than -3 SD from our analysis because the data collectors described them as having very bad health status and the measurements seemed plausible. This left 723 mother-infant pairs to be included in the analysis.

### Data collection, measurements and handling

We used a structured questionnaire that included 24-hour dietary recall and dietary recall since birth on 35 food and liquid items. It also included questions on breastfeeding in general, pre-lacteal feeding, initiation of breastfeeding, socio-demographic characteristics, water and sanitation, education of mothers and fathers, having brothers and/or sisters, type of work, marital status, immunisation status, primary health care usage for the infants and recent sickness.

Weight and recumbent length were taken according to WHO standardized techniques [16]. Undressed infants were weighed to the nearest 0.1 kg using 25 kilogram (kg) portable Salter spring scales and recumbent length was measured to the nearest 0.1 cm. Validation of instruments and measurements and random auditing were done on a daily basis.

Data were entered using EpiData 3.0 and analysed using SPSS 15.0.1 and STATA 9.2. Anthropometric indices were generated using WHO Anthro 2005 software .

### Definitions and analysis

Children's health and nutritional status can be assessed by evaluating their anthropometric data using the z-scores for weight-for-length (WLZ), length-for-age (LAZ) and weight-for-age (WAZ). Anthropometric status was assessed using the WHO Child Growth Standards [12]. Wasting was defined as WLZ less than -2, stunting as LAZ less than – 2 and underweight as WAZ less than – 2 [16]. Pre-lacteal feeding was defined as any liquid or food item given to the infants during the first three postnatal days. The variable initiation of breastfeeding was categorised as follows: (1) within 2 hours (this encompassed 'immediately' and 'within 2 hours'); (2) within the 1^st ^day; and (3) after the 1^st ^day. Infant feeding mode was categorised on the basis of WHO definitions and recommendations: (1) exclusively breastfed (EBF) infants were fed on breast milk only; (2) mixed-fed (MF) infants were fed on breast milk and other feeds; and (3) replacement-fed (RF) infants did not receive breast milk [13,17]. For our analysis we merged the latter two categories. The mode of feeding was assessed according to 24-hour recall. The mothers were asked what they thought about colostrum on an ordinal scale ranging from good (1) to bad (5). Actual practices regarding colostrum were not covered in the questionnaire. Fewer than half the infants were weighed at birth. Although the birth weight written on the health chart was omitted from further analysis, the fact that 'the infants were weighed at birth' was recorded. Completed immunisation status according to the national programme within one month after the recommended time qualified as 'adequate immunisation.' Immunisation that was either incomplete or delayed by more than a month according to the national programme was classed as 'not adequately immunised'.

Household wealth was assessed by constructing an index using principal components analysis. The first component, which explains most of the variance in the observed set of variables, is expected to reflect an unobserved dimension, and in the given model 'wealth.' This method was established in epidemiology during the 1990s and has been used as a proxy for wealth assessment in the literature [18-20]. The variables included in our factor analysis were the following: (1) assets: radio, television, telephone and cupboard; (2) housing material for roof, walls and floor; and (3) toilet, source of light, source of cooking and source of water. Ownership of land was kept separate [6]. The first component explained 40% of the variance. The regression scores from the first component were used to create an index that was divided into quintiles and then grouped as the top 20%, the middle 40% and the bottom 40%. This categorization was chosen in line with Filmer and Pritchett's work and applies where the majority of the population are described as poor according to most definitions and only a smaller proportion of the population possess items associated with higher living standard (e.g. car, indoor tap water, tiled roof, etc.) [18]. Since more than a hundred villages were selected within six sub-counties, three rural and three urban, we wanted to control for potential differences in wealth between these sub-counties [21]; we knew from qualitative reports (unpublished data) that they differ in economic profile. We therefore ranked the sub-counties according to mean wealth based on the ranked regression scores from the household wealth assessment. We paired the top, the mid and the bottom sub-counties from the urban and rural areas, respectively.

**Figure 1:**
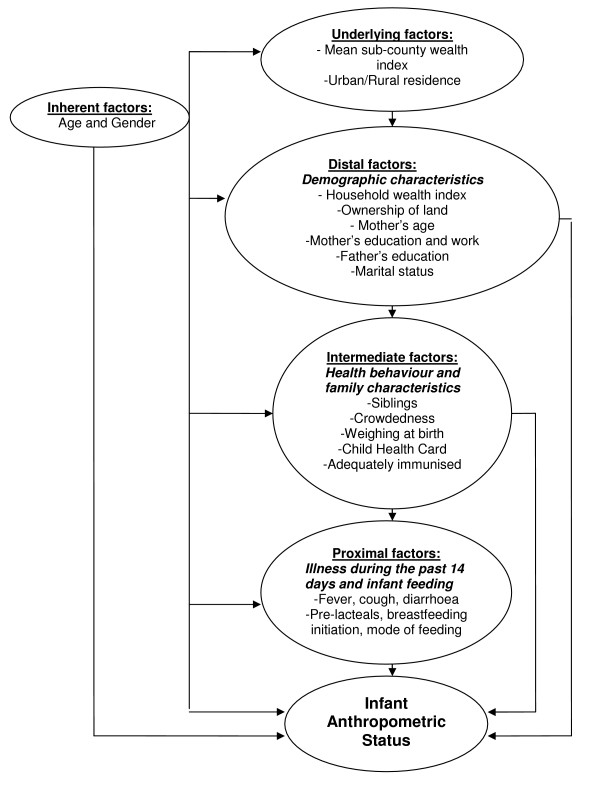
**The conceptual hierarchical framework used as the basis for controlling the factors in multivariate analysis**.

Dependent dichotomous variables were constructed using WLZ and LAZ less than -2 as cut-off value. Potential associated determinants were assessed by crude and adjusted analyses, the latter conducted according to a conceptual hierarchical framework. This framework was established in paediatric epidemiology by Mosley and Chen in 1984 for the study of infant mortality, and refined and elaborated upon by Victora et al. in 1997 with the example of diarrhoea; and similar models have been used by Chopra and Wamani, among others, for the study of anthropometry [6,22-24]. The hierarchical order of assessment of the factors controlled for acknowledged the conceptual basis of each factor's interrelationships with previous ones, preceding exclusion on the basis of significance levels alone, which is common in adjusted analysis. It is argued that this better reflects the true relationships among the factors included in the model. Figure 1 shows the factors controlled for in a hierarchical order for WLZ and LAZ dichotomised on < -2. Age and gender were considered inherent factors and were controlled for in all models and at each stage irrespective of the significance level. The underlying factors, sub-county wealth and urban/rural status, together with age and gender, constituted the first stage. Age and gender remained in the model together with significant underlying factors, while distal factors were added to constitute the second stage. Then intermediate and proximate variables were added as described for stage two, constituting stages three and four. All variables added in the first to the fourth stages remained in the model as long as they were significant at the p < 0.05 level. The odds ratios (OR) from this model for factors associated with stunting were presented: all significant factors are given in addition to inherent factors at all stages.

Anthropometric mean indices were assessed with respect to pre-lacteal feeding, mode of feeding and household wealth. Linear regression analysis was performed for all the explanatory factors before factors were selected for the adjusted means in order to identify potential confounding between feeding practices and anthropometric z-scores, and household wealth and anthropometric z-scores. The following factors were included from Figure 1 with respect to feeding practices and mean z-scores: (1) inherent factors (both); (2) underlying factors (both); (3) distal factor: household wealth and mother's education; (4) intermediate factors (all); and (5) proximate factors: fever, cough and diarrhoea. The relationship between household wealth and mean anthropometric indices was also investigated and was adjusted for all inherent, intermediate and proximate factors. The general linear model (GLM) multivariate analysis in SPSS 15 was used to provide descriptive adjusted means. The crude relationships between household wealth and z-scores were illustrated and the adjusted mean indices were given.

All 723 infants up to one year of age were included in all data analyses. Because exclusive breastfeeding is recommended only for those under six months, we looked at this group in particular (n = 412) and did sub-group analyses of it. Descriptive statistics were used to record the frequencies of those who were wasted, stunted and underweight. Mean WLZ, LAZ and WAZ were assessed for boys and girls. Means with 95% confidence intervals, t-tests and one-way analysis of variance (ANOVA) were used for continuous variables and a chi-square test for categorical data. The significance level was set to *p *< 0.05. The cluster effect arising from the sample design was accounted for by setting the village number (113) as the sampling unit using the 'svyset' command in Stata [25].

### Ethics

Approval of the study was granted by Makerere University Faculty of Medicine Ethics and Research Committee, the Uganda National Council for Science and Technology and the Regional Committee for Medical Research Ethics, Western Norway. Informed consent was obtained from all the study participants, and permission was also obtained from the local administrative units.

## Results

**Figure 2:**
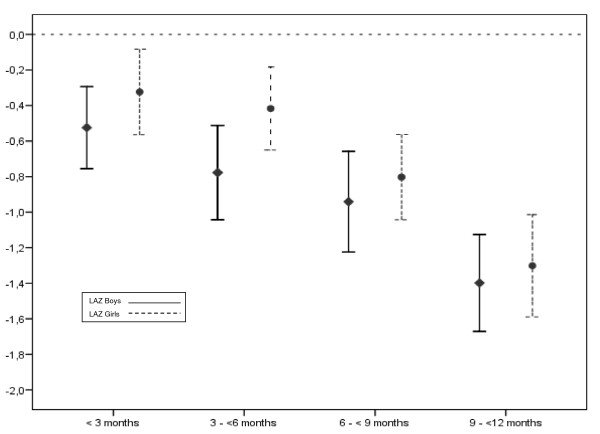
**The mean LAZ with 95% confidence intervals of the mean for boys (straight line) and girls (dashed line) by age categories (< 3 months, 3 -< 6 months, 6 -< 9 months and 9 -< 12 months)**.

The mean WLZ was 0.04 (95% CI -0.07 to +0.14), the mean LAZ was -0.76 (95% CI -0.88 to -0.65) and the mean WAZ was -0.49 (95% CI -0.59 to -0.39) (Table 1). Boys had significantly lower LAZ than girls, and the mean LAZ decreased with age (Figure 2). Among the infants who were stunted, 58.7% were boys and 41.3% girls. There was no significant difference between boys and girls in mean WLZ or WAZ. The proportions of wasted, stunted and underweight children were 4.2%, 16.7% and 9.7%, respectively. The proportion of children with z-scores between -2 and 2 were 91.2% for WLZ, 82.3% for LAZ and 88.4% for WAZ.

**Table 1:** Mean anthropometric indices and comparison according to sex

	**All infants aged 0–11 months (n = 723)**			A subset of infants aged 0–5 months (n = 412)		
	WLZ Mean (95% CI)	LAZ Mean (95% CI)	WAZ Mean (95% CI)	WLZ Mean (95% CI)	LAZ Mean (95% CI)	WAZ Mean (95% CI)
All infants (n = 723)	+0.04 (-0.07 to +0.14)	-0.76 (-0.88 to -0.65)	-0.49 (-0.59 to -0.39)	+0.01 (-0.11 to +0.14)	-0.52 (-0.66 to -0.38)	-0.41 (-0.53 to -0.30)
Boys (n = 378)	+0.07 (-0.06 to +0.20)	-0.85 (-1.00 to -0.72)	-0.52 (-0.66 to -0.39)	+0.06 (-0.10 to +0.21)	-0.65 (-0.86 to -0.43)	-0.47 (-0.63 to -0.30)
Girls (n = 345)	-0.004(-0.13 to +0.14)	-0.66 (-0.80 to -0.51)	-0.45 (-0.58 to -0.33)	-0.04(-0.22 to 0.14)	-0.37 (-0.54 to -0.20)	-0.35 (-0.50 to -0.20)
t-test *p*	NS*	0.038	NS*	NS*	0.03	NS

*Not significant

The mean ages for introducing feeds were one month for water-based liquids and two months for complementary feeds. More than half the infants (57%) had received pre-lacteal feeding, predominantly water-based feeds, which skewed the median age for introducing water-based liquids to three days. The latest time-point reported for introducing feeds other than breast milk was seven months. Breastfeeding was practised by 98% at the time of the interview. Thirty-eight percent of the mothers perceived colostrum as good and 35% perceived it as bad, and the remaining mothers had less polarised views.

### Baseline characteristics

The baseline characteristics are presented together with the crude ORs (95% CI) for the dichotomized variables WLZ and LAZ, with < -2 as cut-off value (Table 2). The only unadjusted factor associated with wasting (WLZ < -2) was having had diarrhoea during the previous 14 days, with a two-fold increase in odds ratio (OR 2.16, 95% CI 1.02–6.61).

**Table 2:** Baseline characteristics and crude odds ratios (OR) from logistic regression analysis for wasting (WLZ < -2) and stunting (LAZ < -2)

**N-total: 723**	**n**	**(%)**	**Crude** **WLZ < -2 OR**	**95% CI**	**Crude** **LAZ < -2 OR**	**95% CI**
**Inherent factors**						
Gender of infant						
Girl	345	47.7	1.26	0.69–2.31	0.73	0.48–1.11
Age constant (0–11 cont)	723		0.98	0.86–1.11	1.13	1.07–1.20^a^
**Underlying factors**						
Mean sub-county wealth						
Highest	238	32.9	1.00		1.00	
Mid	279	38.6	1.65	0.68–3.98	0.99	0.62–1.60
Lowest	206	28.5	0.50	0.16–1.61	1.48	0.94–2.34
Urban/Rural status						
Rural status	398	55.1	1.67	0.66–4.22	1.41	0.95–2.08
**Distal factors**						
Houshold wealth						
Top 20%	143	19.8	1.00		1.00	
Mid 40%	291	40.2	1.76	0.54–5.70	1.59	0.82–3.10
Lowest 40%	189	40.0	1.51	0.48–4.71	2.62	1.38–4.98^b^
Owning land						
Yes	557	77.0	1.06	0.40–2.81	1.45	0.86–2.45
Mother's age						
24 and younger	373	51.6	1.00		1.00	
25 and older	343	47.4	0.72	0.36–1.43	0.82	0.57–1.16
Marital status						
Traditional	581	80.4	1.00		1.00	
Formal marriage	77	10.7	0.87	0.25–2.94	1.09	0.58–2.08
Single, separated, divorced, widowed	65	9.0	0.33	0.04–2.60	1.91	0.99–3.66
Mother has additional job						
Yes	131	18.3	0.50	0.14–1.76	0.63	0.37–1.10
Mother's education						
Full primary and less (≤ 7 years)	482	66.7	1.00		1.00	
Lower secondary and more (≥ 8 years)	228	31.5	0.90	0.42–1.95	0.78	0.52–1.18
Father's education						
Full primary and less (≤ 7 years)	351	48.5	1.00		1.00	
Lower secondary and more (≥ 8 years)	266	36.8	1.14	0.52–2.47	0.70	0.43–1.13
**Intermediate factors**						
Infant has siblings						
Yes	549	75.9	0.99	0.41–2.38	0.66	0.44–1.00
Crowdedness; number of people per room						
≤ 3	114	15.8	1.00		1.00	
4–5	231	32.0	0.90	0.31–2.64	0.68	0.38–1.23
6–7	198	27.4	0.37	0.09–1.40	0.68	0.36–1.26
≥ 8	180	24.9	0.95	0.35–2.55	0.96	0.53–1.71
Weighing at birth						
Yes	334	46.2	0.87	0.45–1.70	0.69	0.47–1.00
Child health card						
Yes	501	69.3	0.58	0.25–1.33	0.85	0.55–1.32
Adequately immunised						
Yes	233	32.2	0.63	0.25–1.55	0.68	0.41–1.12
**Proximal factors**						
Fever last 14 days						
Yes	416	57.5	2.25	0.90–6.63	1.37	0.89–2.12
Cough last 14 days						
Yes	564	78.0	1.81	0.66–4.97	1.76	1.04–2.98^c^
Diarrhoea last 14 days						
Yes	266	36.8	2.16	1.02–6.61^c^	1.26	0.86–1.88
Pre-lacteals						
Yes	414	57.3	1.78	0.73–4.37	1.26	0.84–1.91
Initiation of breastfeeding						
Within 2 hours	364	50.3	1.00		1.00	
Within the 1^st ^day	128	17.7	1.07	0.39–2.92	1.06	0.56–1.99
After the 1^st ^day	193	26.7	0.81	0.33–2.06	1.27	0.80–2.03
Mixed feeding/Replacement feeding						
Yes	608	84.1	1.73	0.53–5.67	4.23	1.81–9.93^a^

^a ^p ≤ 0.001, ^b ^p ≤ 0.01, ^c ^p ≤ 0.05

The crude factors associated with stunting were: (1) age; (2) lowest household wealth; (3) coughing during the previous 14 days; and (4) being mixed or replacement fed. The mother's marital status was borderline significant in the crude analysis, with a two-fold increase in odds ratio for being stunted if the mother was single, divorced, separated or widowed. In contrast, having brothers and/or sisters was protective (OR 0.66, 95% CI 0.44–1.00).

### Factors associated with stunting and wasting according to the conceptual hierarchical framework

The conceptual hierarchical framework was the basis for adjusted logistic regression analysis. No determinants were found to be significantly associated with wasting when all infants, n = 723, were included. In a sub-group analysis of infants under six months, n = 412, pre-lacteal feeding was significantly associated with wasting (OR 4.63, 95% CI 1.11–19.23).

Stunting was associated with many factors including all infants, n = 723 (Table 3). According to the conceptual hierarchical framework, age and gender were present in the regression model throughout the four stages: gender was found to be associated with stunting at stages two and three, and age throughout the model. In the first stage, underlying factors were controlled for: being in the least wealthy sub-counties was associated with stunting (OR 1.64, 95% CI 1.00–2.71). At stage two, distal factors were added: lowest household wealth was significantly associated with stunting and remained in the model at stages three and four with a three-fold increase in odds ratio (OR at entry of model: 3.50, 95% CI 1.57–7.78). Having brothers and/or sisters was also a protective factor in the adjusted model. Mixed feeding or replacement feeding was the only proximate factor significantly associated with stunting in the adjusted analysis (OR 2.71, 95% CI 1.02–7.13). A sub-group analysis was also done for stunting among those under six months: mixed feeding or replacement feeding was then associated with stunting with a three-fold increase in OR (OR 3.35, 95% CI 1.19–9.45). Having brothers and/or sisters was the only other baseline characteristic found to be associated with stunting in the sub-group analysis.

**Table 3:** Adjusted logistic regression for LAZ < -2.

**Adjusted LAZ < -2, total: 723**	**LAZ < -2 OR**	**95% CI**	**LAZ < -2 OR**	**95% CI**	**LAZ < -2 OR**	**95% CI**	**LAZ < -2 OR**	**95% CI**
	**Stage 1**		**Stage 2**		**Stage 3**		**Stage 4**	
**Inherent factors**								
Gender of infant	0.70	0.46–1.07	0.66	0.41–1.08	0.63	0.41–0.97^c^	0.62	0.39–0.97^c^
Girl	1.14	1.08–1.21^a^	1.15	1.07–1.22^a^	1.17	1.10–1.25^a^	1.10	1.03–1.19^b^
Age constant (0–11 cont)								
**Underlying factors**								
Mean sub-county wealth								
Highest	1.00							
Mid	1.06	0.65–1.72						
Lowest	1.64	1.00–2.71^c^						
**Distal factors**								
Houshold wealth								
Top 20%			1.00		1.00		1.00	
Mid 40%			1.65	0.74–3.68	1.71	0.87–3.36	1.30	0.62–2.73
Lowest 40%			3.50	1.57–7.78^b^	3.10	1.56–6.15^a^	2.70	1.39–5.28^b^
**Intermediate factors**								
Infant has siblings								
Yes					0.43	0.24–0.79^b^	0.60	0.38–0.94^c^
**Proximal factors**								
Mixed feeding/Replacement feeding								
Yes							2.71	1.02–7.13^c^

^a ^p ≤ 0.001, ^b ^p ≤ 0.01, ^c ^p ≤ 0.05

Significant factors in addition to age and gender presented according to the hierarchical conceptual framework in figure 1.

### Anthropometric indices

**Figure 3:**
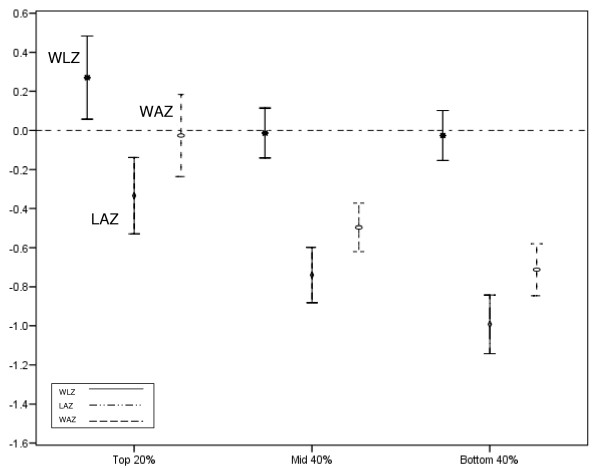
**WLZ, LAZ and WAZ with 95% confidence intervals of the mean by wealth category**.

The adjusted mean anthropometric indices were investigated with respect to pre-lacteal feeding, mode of feeding and household wealth. Pre-lacteal feeding was inversely associated with WLZ and WAZ (regression-coefficient -0.20, p = 0.023 and -0.22, p = 0.012, respectively) (Table 4a). Mixed feeding or replacement feeding was inversely associated with LAZ (regression-coefficient -0.32, p = 0.03) (Table 4b). Household wealth was associated with LAZ and WAZ: after adjusting for all inherent, intermediate and proximate factors the adjusted regression-coefficient between the top and bottom wealth categories was -0.58, p < 0.001 for LAZ, and -0.49, p < 0.001 for WAZ (Table 5). Crude decreasing anthropometric trends were seen with decreasing household wealth (Figure 3).

**Table 4:** Adjusted mean anthropometric indices according to pre-lacteal feeding (4a) and mode of feeding (4b) status

4a:			
**All infants aged 0–11 months (n = 723)**			

***Pre-lacteal feeding***	**Mean WLZ**	**Mean LAZ**	**Mean WAZ**
Pre-lacteals given	-0.03 (-0.15 to +0.08)	-0.82 (-0.94 to -0.70)	-0.58 (-0.70 to -0.47)
Pre-lacteals not given	+0.17 (+0.04 to +0.30)	-0.70 (-0.84 to -0.56)	-0.36 (-0.49 to -0.23)
*Adjusted regression-coefficient*	*-0.20, p = 0.023*	-0.12, NS*	*-0.22, p = 0.012*

4b:			

**All infants aged 0–11 months (n = 723)**			

***Mode of feeding***	**Mean WLZ**	**Mean LAZ**	**Mean WAZ**
Mixed and replacement fed	0.06 (-0.03 to +0.15)	-0.82 (-0.92 to -0.72)	-0.53 (-0.62 to -0.43)
Exclusively breastfed	0 (-0.24 to +0.24)	-0.50 (-0.76 to -0.24)	-0.28 (-0.52 to -0.03)
*Adjusted regression-coefficient*	0.06, NS*	*-0.32, p = 0.03*	0.25, p = NS*

*Not significant

** The mean WLZ, LAZ and WAZ were adjusted for the same factors in general linear multivariate analysis (GLM): Age, gender, sub-county wealth, urban-rural status, household wealth, mother's education, siblings, crowdedness, having a child health card, weighing at birth, vaccination status, fever, cough, diarrhoea

**Table 5:** Adjusted mean anthropometric indices according to household wealth status

**All infants aged 0–11 months (n = 723)**			
**Houshold wealth**	**Mean WLZ**	**Mean LAZ**	**Mean WAZ**
Top 20%	0.15 (-0.05 to +0.35)	-0.42 (-0.64 to -0.20)	-0.19 (-0.40 to +0.01)
Mid 40%	0.03 (-0.11 to +0.16)	-0.69 (-0.84 to -0.55)	-0.44 (-0.58 to -0.31)
Lowest 40%	0.009 (-0.13 to +0.15)	-1.00 (-1.15 to -0.85)	-0.68 (-0.82 to -0.54)
*Adjusted regression-coefficient*	0.141, p = NS*	*-0.58, p < 0.001*	*-0.49, p < 0.001*

*Not significant

** The mean WLZ, LAZ and WAZ were adjusted for all inherent, intermediate and proximal factors (table 1) in general linear multivariate analysis (GLM).

## Discussion

The aim of this study was to describe growth patterns in Eastern Uganda using the WHO Child Growth Standards, and to investigate factors that might be associated with growth outcomes, including infant feeding practices. From the results it is clear that lower anthropometric status was associated with household wealth; in particular, the lowest household wealth was related to stunting with a threefold increase in OR. This study showed an increased prevalence of stunting with age and a significant difference between boys and girls in length-for-age: boys were more vulnerable. No factors were significantly associated with wasting in the adjusted analysis. This was probably due to the low proportion of wasted children. Adjusted mean differences showed that pre-lacteal feeding was associated with lower WLZ. Mixed feeding or replacement feeding was associated with lower LAZ after adjustment for other explanatory factors.

The cross-sectional design of the study entails certain limitations. First, only associations can be described; causal relationships cannot be established. It would for example be tempting to say that mixed feeding or replacement feeding leads to worse length-for-age outcomes. Theoretically, the converse might be true (reverse causality) [26,27]: those with low LAZ are more likely to receive supplementary feeds in addition to breast milk in order to boost their growth. Second, only the surviving participants from the catchment area are included in the study, and that in itself entails an inbuilt selection bias. We could not obtain information about those who were dead, hospitalised or travelling. We might, for example, speculate that if dead infants were included, other more striking factors than household wealth and feeding would emerge as hindrances to growth, especially infectious diseases. Third, as the study was questionnaire-based, questions that require a good memory or might be sensitive were more vulnerable to recall bias or to socially desirable answers. Fourth, the reproducibility of the answers and measurements cannot be assessed for reasons of feasibility. Stringent training, frequent validation of instruments and procedures together with random auditing do not guarantee objectivity, but they are tools by which errors are minimised and internal validity strengthened. Fifth, certain aspects of the questionnaire could have been more specific: the most common way to record initiation of breastfeeding is "within the first hour," whereas we recorded "(1) immediately and (2) within the first two hours" etc. This reduces comparability with other studies. Likewise, actual practices regarding colostrum could have been covered in greater detail as we were focusing on early infant feeding practices. When these limitations are taken into account, we find the results plausible as they are consistent with existing literature in this field. Other Ugandan anthropometric studies also report findings that indicate differences between boys and girls using WHO Child Growth standards [28], and the importance of recommended infant feeding practices and socio-economic factors as closely linked with health outcomes [7,29]. A recently published community study of children from 6 to 59 months in Bundibugyo District, Western Uganda, found wasting and stunting rates of 3 and 44%, respectively. The authors call for public health messages that will lead to decreased stunting among children [30]. Our findings that wasting and stunting rates were 4 and 17%, respectively, among infants only, support the need for public health action to improve the nutritional status of the youngest children [4]. Education of mothers did not turn out to be a significant factor in the adjusted analysis in our study, but education should not be underestimated as it clearly correlates with socio-economic status and health behaviour, as shown in other studies from Uganda [31].

Those who had received pre-lacteal feeds tended to have poorer weight-for-length outcomes according to the adjusted means in the multivariate general linear model. Various explanations are possible. First, there may be a physiological explanation [32]. The possibly harmful effect of pre-lacteal feeding and therefore late initiation of breastfeeding could have on the establishment of successful breastfeeding was described in Ugandan literature nearly two decades ago. The result, described as "insufficient milk syndrome", will further impair development and growth [33]. The negative effect of pre-lacteal feeding on the establishment of successful breastfeeding has also been described in studies from Latin America [34]. Second, there may be a pathological explanation: if the pre-lacteal feeds received by the infants induced sickness, reduced growth and symptoms of failure to thrive might become apparent early [35,36]. And third, a behavioural and educational explanation is possible: integrated management of childhood illness (IMCI) was launched in Uganda in 1995 [37]. IMCI gives us ten steps to achieving successful breastfeeding; pre-lacteal feeding is discouraged (step 6) and initiation of breastfeeding within half an hour after birth is promoted (step 4) [38]. Since step 6 was violated by more than half and step 4 by two-thirds of the participants in our study, it is possible that many other breastfeeding-supportive IMCI messages also are omitted from the health education of the mothers [37]. Alternative infant feeding practices with poor nutritional value might explain some of the differences in growth [7]. The reasons for which health workers might give or suggest pre-lacteal feeds have been described elsewhere [39]. It is also possible that messages related to early infant feeding practices are misconceived by both health workers and mothers in this Ugandan setting.

We used 'weighing at birth,' 'having a child health card' and 'being adequately immunised' as indicators of 'contact with primary health care units.' No association was seen with wasting and stunting in the crude or adjusted analyses. These variables were included when the mean anthropometric indices were adjusted for feeding practices and household wealth because they were associated with WLZ and LAZ in the linear regression. Improved anthropometric status could be attributable to better contact with respective health units because of improved health care, including nutritional messages with growth-protective value, actual treatment and immunisation. Mothers who delivered at health units or took their new-borns for weighing could also have been more trained and empowered. An association was seen between the ranking of sub-counties and stunting and this could possibly be explained by access to health services within the respective sub-counties. The present study was not designed to address this, but recent Indian studies suggest such relationships [40].

A more meaningful term than 'feeding mode' would have been a constructed feeding index where infant phase-specific preferred feeding gave higher credits and non-recommended feeding lower credits. Two comprehensive nutritional studies from Africa in which such an index was utilised have been published recently [41,42]. Unfortunately, our study was not designed to utilise their suggested model. What this and the previous study from Mbale could detect [13] was that complementary feeding practices were inadequate and infant stunting rates were far too high. This should in itself raise public health concerns. We also recommend that more comprehensive designs should be utilised in future Ugandan nutritional studies in order to achieve more infant phase-specific information. The Uganda Demographic and Health Survey (UDHS) reports high breastfeeding rates, reduced exclusive breastfeeding practices in the first half of infancy combined with delayed provision of recommended complementary foods of insufficient amount and diversity [9]. Uganda has recently been presented as an example of different successful interventions [43], but striking needs are still seen in nutrition and delivery of health care services [9].

## Conclusion

This study describes the growth patterns of a randomly-selected community-based infant population in Eastern Uganda and factors explaining these patterns. It utilised a conceptual hierarchal framework and found associations with growth at all levels of the models, supporting the view that child growth is multi-dimensional. Household wealth showed the strongest association with stunting. These findings are in line with earlier anthropometric descriptions from Western and Northern Uganda including older children, and this study indicated no changes or improvements [6,7,30,31]. Impaired growth is associated with inequity and lack of empowerment. There may be an urgent need for holistic approaches towards the infants, families and communities in order to bridge some of the gaps [3], and there must be a focus on the youngest children in nutrition programmes to avoid long-term effects [5]. Early initiation of breastfeeding, avoidance of pre-lacteal feeding, exclusive breastfeeding for six months and improved feeding practices in the second half of infancy are important and clear public health messages, which need to be communicated to pregnant women in a sound and respectful way.

## Competing interests

The authors declare that they have no competing interests.

## Authors' contributions

IE was active during the design, implementation, analysis and writing. HW and CK contributed to the design and analysis. JKT and TT initiated the study and contributed throughout the whole process of design, implementation, analysis and co-writing. All authors read and approved the final manuscript.

## Pre-publication history

The pre-publication history for this paper can be accessed here:



## References

[B1] Black RE, Allen LH, Bhutta ZA, Caulfield LE, de Onis M, Ezzati M, Mathers C, Rivera J (2008). Maternal and child undernutrition: global and regional exposures and health consequences. Lancet.

[B2] Edmond KM, Zandoh C, Quigley MA, Amenga-Etego S, Owusu-Agyei S, Kirkwood BR (2006). Delayed breastfeeding initiation increases risk of neonatal mortality. Pediatrics.

[B3] Martin L, Hossain SMM, Casanovas C, Guyon A (2008). Learning from Large-Scale community-Based Programs to Improve Breastfeeding Practices.

[B4] Bhutta ZA, Ahmed T, Black RE, Cousens S, Dewey K, Giugliani E, Haider BA, Kirkwood B, Morris SS, Sachdev HP (2008). What works? Interventions for maternal and child undernutrition and survival. Lancet.

[B5] Victora CG, Adair L, Fall C, Hallal PC, Martorell R, Richter L, Sachdev HS (2008). Maternal and child undernutrition: consequences for adult health and human capital. Lancet.

[B6] Wamani H, Astrom AN, Peterson S, Tumwine JK, Tylleskar T (2006). Predictors of poor anthropometric status among children under 2 years of age in rural Uganda. Public Health Nutr.

[B7] Kikafunda JK, Walker AF, Collett D, Tumwine JK (1998). Risk factors for early childhood malnutrition in Uganda. Pediatrics.

[B8] Bridge A, Kipp W, Jhangri GS, Laing L, Konde-Lule J (2006). Nutritional status of young children in AIDS-affected households and controls in Uganda. Am J Trop Med Hyg.

[B9] (2006). Uganda Demographic and Health Survey 2006.

[B10] Maleta K, Virtanen SM, Espo M, Kulmala T, Ashorn P (2003). Childhood malnutrition and its predictors in rural Malawi. Paediatr Perinat Epidemiol.

[B11] Kourtis AP, Jamieson DJ, de Vincenzi I, Taylor A, Thigpen MC, Dao H, Farley T, Fowler MG (2007). Prevention of human immunodeficiency virus-1 transmission to the infant through breastfeeding: new developments. Am J Obstet Gynecol.

[B12] Garza C, de Onis M (2004). Rationale for developing a new international growth reference. Food Nutr Bull.

[B13] Engebretsen IM, Wamani H, Karamagi CA, Semiyaga N, Tumwine JK, Tylleskar T (2007). Low adherence to exclusive breastfeeding in Eastern Uganda: a community-based cross-sectional study comparing dietary recall since birth with 24-hour recall. BMC Pediatr.

[B14] Karamagi CA, Tumwine JK, Tylleskar T, Heggenhougen K (2006). Antenatal HIV testing in rural eastern Uganda in 2003: incomplete rollout of the prevention of mother-to-child transmission of HIV programme?. BMC Int Health Hum Rights.

[B15] WHO Child Growth Standards Length/height-for-age, weight-for-age, weight-for-length, weight-for-height and body mass index-for-age Methods and development. http://www.who.int/childgrowth/publications/ca_symposium_fieldtesting/en/index.html.

[B16] (1995). Physical status: The use and interpretation of anthropometry.

[B17] HIV and Infant Feeding Guidelines for decision makers 2003. http://www.who.int/child-adolescent-health/New_Publications/NUTRITION/HIV_IF_DM.pdf.

[B18] Filmer D, Pritchett LH (2001). Estimating wealth effects without expenditure data – or tears: an application to educational enrollments in states of India. Demography.

[B19] Indepth-Network (2005). Measuring health equity in small areas – Findings from demographic surveillance systems.

[B20] Rutstein SO, Johnson K (2004). The DHS Wealth Index. ORC Macro, DHS Comparative Reports 6.

[B21] Rajaratnam JK, Burke JG, O'Campo P (2006). Maternal and child health and neighborhood context: the selection and construction of area-level variables. Health Place.

[B22] Chopra M (2003). Risk factors for undernutrition of young children in a rural area of South Africa. Public health nutrition.

[B23] Mosley WH, Chen LC (1984). An analytical framework for the study of child survival in developing countries. Population and Development Review.

[B24] Victora CG, Huttly SR, Fuchs SC, Olinto MT (1997). The role of conceptual frameworks in epidemiological analysis: a hierarchical approach. Int J Epidemiol.

[B25] Bennett S, Woods T, Liyanage WM, Smith DL (1991). A simplified general method for cluster-sample surveys of health in developing countries. World Health Stat Q.

[B26] Bahl R, Frost C, Kirkwood BR, Edmond K, Martines J, Bhandari N, Arthur P (2005). Infant feeding patterns and risks of death and hospitalization in the first half of infancy: multicentre cohort study. Bull World Health Organ.

[B27] León-Cava N, Lutter C, Ross J, Martin L (2002). Quantifying the Benefits of Breastfeeding: A Summary of the Evidence.

[B28] Wamani H, Astrøm AN, Peterson S, Tumwine JK, Tylleskär T (2007). Boys are more stunted than girls in sub-Saharan Africa: a meta-analysis of 16 demographic and health surveys. BMC Pediatrics.

[B29] Wamani H, Astrøm AN, Peterson S, Tylleskär T, Tumwine JK (2005). Infant and young child feeding in western Uganda: knowledge, practices and socio-economic correlates. Journal of Tropical Pediatrics.

[B30] Jilcott SB, Masso KL, Ickes SB, Myhre SD, Myhre JA (2007). Surviving but not quite thriving: anthropometric survey of children aged 6 to 59 months in a rural Western Uganda district. J Am Diet Assoc.

[B31] Wamani H, Tylleskär T, Astrøm AN, Tumwine JK, Peterson S (2004). Mothers' education but not fathers' education, household assets or land ownership is the best predictor of child health inequalities in rural Uganda. International Journal for Equity in Health.

[B32] Lawrence R, Lawrence R (1999). Breastfeeding A guide for the medical profession.

[B33] Mukasa GK (1992). A 12-month lactation clinic experience in Uganda. Journal of Tropical Pediatrics.

[B34] Pérez-ascamilla R, Segura-Millán S, Canahuati J, Allen H (1996). Prelacteal feeds are negtively associated with breast-feeding outcomes in Honduras. J Nutr.

[B35] Arifeen SE, Black RE, Caulfield LE, Antelman G, Baqui AH (2001). Determinants of infant growth in the slums of Dhaka: size and maturity at birth, breastfeeding and morbidity. Eur J Clin Nutr.

[B36] Lawn JE, Cousens S, Zupan J (2005). 4 million neonatal deaths: when? Where? Why?. Lancet.

[B37] Karamagi CA, Lubanga RG, Kiguli S, Ekwaru PJ, Heggenhougen K (2004). Health Providers' Counselling of Caregivers in the Integrated Management of Childhood Illness (IMCI) Programme in Uganda. African Health Sciences.

[B38] Evidence for the ten steps to successful breastfeeding. http://www.who.int/child-adolescent-health/publications/NUTRITION/WHO_CHD_98.9.htm.

[B39] Akuse RM, Obinya EA (2002). Why healthcare workers give prelacteal feeds. European Journal of Clinical Nutrition.

[B40] Das J, Hammer J (2007). Location, location, location: residence, wealth, and the quality of medical care in Delhi, India. Health Aff (Millwood).

[B41] Moursi MM, Martin-Prevel Y, Eymard-Duvernay S, Capon G, Treche S, Maire B, Delpeuch F (2008). Assessment of child feeding practices using a summary index: stability over time and association with child growth in urban Madagascar. Am J Clin Nutr.

[B42] Sawadogo PS, Martin-Prevel Y, Savy M, Kameli Y, Traissac P, Traore AS, Delpeuch F (2006). An infant and child feeding index is associated with the nutritional status of 6- to 23-month-old children in rural Burkina Faso. J Nutr.

[B43] Parkhurst JO (2008). "What worked?": the evidence challenges in determining the causes of HIV prevalence decline. AIDS Educ Prev.

